# Adhesive Ability of Different Oral Pathogens to Various Dental Materials: An *In Vitro* Study

**DOI:** 10.1155/2022/9595067

**Published:** 2022-08-01

**Authors:** Yan Tu, Shuli Deng, Yuan Wang, Xiaolong Lin, Zhenyu Yang

**Affiliations:** Department of Endodontics, Stomatology Hospital, School of Stomatology, Zhejiang University School of Medicine, Zhejiang Provincial Clinical Research Center for Oral Diseases, Key Laboratory of Oral Biomedical Research of Zhejiang Province, Cancer Center of Zhejiang University, Hangzhou 310000, China

## Abstract

**Introduction:**

In dental treatments, the reason for secondary caries and the failure of root canal treatment is the microbial infection, which concerns most dentists. The challenge of how to reduce the number of bacteria at the filling materials and the number of residual bacteria in the root canal has become a research hotspot. In this study, the bacterial adhesion properties of several common dental materials were compared to provide a theoretical basis for the selection of antibacterial properties of dental materials. *Methodology*. Three commonly used dental restorative materials and five sealers in root canal treatment were selected. Each material block was immersed in the corresponding supragingival (*Streptococcus mutans* and *Actinomyces viscosus*) or subgingival (*Porphyromonas gingivalis* and *Enterococcus faecalis*) bacterial solution and cultured under anaerobic conditions at 37°C for 2, 4, 6, 8, 12, 16, 20, and 24 h. The adhesion of bacteria was observed, and the number of different bacteria adhering to various material model disks was calculated at different time intervals under a scanning electron microscope. The adherent CFU load of the materials was determined by colony counting.

**Results:**

*Streptococcus mutans* and *Actinomyces viscosus* exhibited the strongest adhesion ability to the resin material blocks. *Porphyromonas gingivalis* and *Enterococcus faecalis* exhibited the highest adhesion ability to the AH-Plus sealer block.

**Conclusions:**

In dental treatments, dental materials should be selected based on the chemical, physical, and biological properties of materials. In addition, it is necessary to develop new antibacterial dental materials.

## 1. Introduction

Biofilms can be formed on almost all surfaces exposed to the natural environment [1]. Undisputedly, a classic biofilm structure can form in the oral cavity. Currently, controlling biofilm formation in the oral cavity is a long-term goal. Biofilms can cause caries and periodontal diseases, as hard and soft tissue conditions in the oral cavity, respectively [2]. Although biofilm formation on the surface of oral biomaterials might appear harmless, the hazard is similar to that of periodontal disease and peri-implantitis [3]. Class II restorations that overextend to the gingival margin are prone to bacterial colonization, endangering gingival health [4–6]. The biofilm formed on the composite resin will not only degrade the surface material and affect its hardness [7] but will also help bacteria invade the tooth‒restorative material interface [8], leading to secondary caries [9] and pulp irritation [10]. The biofilms formed during orthodontic treatment can cause enamel demineralization around orthodontic materials, resulting in orthodontic treatment-related complications [11, 12]. Therefore, many researchers have focused on studying new dental materials that attract less biofilm or release antibiotics. The biofilm formed on the composite resin and glass ionomer cement can lead to material denaturation [7] and surface degradation, promoting the formation of more biofilms and further degrading the surface. This mutual influence leads to secondary caries [13]. Icon resin infiltration treatment is an innovative treatment method between remineralization treatment and filling treatment. It achieves the purpose of strengthening demineralized enamel and preventing further demineralization by removing a small amount of tooth tissue. Icon is an innovative product for the minimally invasive treatment of early caries. The material is mainly composed of methyl methacrylate resin matrix without filler particles. It can treat early caries on the adjacent surface and smooth surface. It is a conservative treatment. Paris S and Meyer-Lueckel followed up 22 patients with icon infiltration treatment for 18 months and confirmed that icon treatment can significantly reduce the progression of early caries [14].

Fluoride can be used as a buffer to neutralize the acid generated by bacteria [15] and inhibit the growth of oral bacterial biofilms associated with dental caries [16]. The glass ionomer containing fluoride does form a thin biofilm with low viability (2–3%), reducing the biofilms produced by *Streptococcus mutans* [17] and *Streptococcus sanguinis* [18], probably due to the release of fluorides [19]. However, an *in vitro* study [20] showed that glass ionomer cement containing fluoride did not reduce bacterial and biofilm counts on saliva-soaked surfaces, suggesting that even fluoride was not a decisive factor in controlling biofilm formation. This might not be valid because the fluoride concentration was too low, considering the ratio of the material area to the liquid volume. Due to the large volume of saliva in the oral environment, it is difficult to achieve effective fluoride concentration [21]. However, a study showed that one year after placing 1–6 glass ionomer restorations in the oral cavity, the fluoride level increased 10 folds [22]. However, this also could not predict its antibacterial effect. Therefore, the benefits of fluoride release are believed to be limited to inhibiting demineralization.

Most endodontic treatments fail because the irritants leak into periapical tissues [23–25]. The ideal apical filling material should be able to seal the root canal system and surrounding tissues and be nontoxic, noncarcinogenic, biocompatible with host tissues, insoluble in tissue fluid, and stable [26, 27]. Moreover, its sealing performance should not be affected by moisture. It should be handled easily, be radiopaque, and be easily identified on X-ray films [26]. Mineral trioxide aggregate (MTA) is a biologically active material [28], with hard tissue conductivity [29], hard tissue sensitivity, and biocompatibility. Since the existing endodontic treatment materials do not have these ideal characteristics, in recent years, MTA has been applied as a root apical sealing material, pulp capping agent, apical barrier material, root canal perforation repair material, and root canal sealing material due to its characteristics. iRoot bioceramic materials, which have become popular in recent years, have also been widely accepted because of their excellent biocompatibility.

Many studies are available on the antibacterial properties of MTA. Several investigations have demonstrated the limited antibacterial properties of MTA against some microorganisms [30–33]. A study on facultative and obligate anaerobes showed that [31] MTA had antibacterial effects on some facultative anaerobes, with little impact on strict obligate anaerobes. In contrast, Super EBA and ZOE sealers exhibited antibacterial effects on facultative and obligate anaerobes [31]. An antimicrobial study of MTA found that MTA could not inhibit the growth of *Staphylococcus aureus*, *Enterococcus faecalis*, *Pseudomonas aeruginosa*, *Bacillus subtilis*, *Candida albicans*, wild fungi, and their mixtures [30].

In dental treatments, microbial infections are the leading cause of oral treatment failure; they can also cause secondary caries and the failure of root canal treatment. In this study, we hypothesized that the ability of bacteria to adhere to different dental materials is different. Therefore, the task of how to select various dental materials and develop new dental materials has become a research hotspot. This study aimed to observe differences in adhesion abilities of supragingival and subgingival plaque to different dental restorative materials and root canal sealers *in vitro*. The common supragingival plaque-forming microorganisms, *Streptococcus mutans* and *Actinomyces viscosus*, which could cause secondary caries, and common subgingival plaque-forming microorganisms, *Porphyromonas gingivalis* and *Enterococcus faecalis*, which could cause root canal infection, were selected. The most commonly used supragingival dental restorative materials in the department of endodontics, stomatology hospital, school of stomatology, Zhejiang University school of medicine, including 3M Z350 resin, glass ionomer cement, and icon penetration resin, and the most commonly used subgingival root canal sealing materials in the same hospital (MTA, iRoot-SP, iRoot-BP, iRoot-FS, and AH-Plus) were selected. The differences in the adhesion properties of these bacterial species to different materials and the formation of biofilms were assessed at different time intervals to provide a basis for selecting antibacterial properties of dental materials.

## 2. Methodology

### 2.1. Preparation of Nine Dental Material Model Blocks

Three dental restorative materials, including 3M Z350 resin, glass ionomer cement, icon penetration resin, and five different root canal sealing materials, including MTA, iRoot-SP, iRoot-BP, iRoot-FS, and AH-Plus, were transferred into a prefabricated aseptic mold with a diameter of 10 mm and a height of 1 mm to prepare cylindrical material blocks. After setting, the material block was retrieved, polished, and sterilized by ultraviolet light for 30 min. (see The composition of the dental materials Table 1). 

### 2.2. Bacterial Culture

*Streptococcus mutans* (UA159) was cultured on MS plates and incubated for 24 h at 37°C under 6% CO_2_. *Actinomyces viscosus* (ATCC27044) was cultured on brain-heart infusion (BHI) solid medium blood plates (Oxoid, Basingstoke, UK) and incubated for 48 h at 37°C under anaerobic conditions. *Porphyromonas gingivalis* (ATCC33277) was cultured on BHI solid medium blood plates and incubated for 72 h at 37 °C under anaerobic conditions. *Enterococcus faecalis* (ATCC19433) was cultured on BHI solid medium blood plates and incubated for 48 h at 37°C under anaerobic conditions.

### 2.3. Bacterial Treatment

*Streptococcus mutans* (UA159), *Actinomyces viscosus* (ATCC27044), *Porphyromonas gingivalis* (ATCC33277), and *Enterococcus faecalis* (ATCC19433) strains were washed in sterile phosphate-buffered saline (PBS) solution and centrifuged at 150 g for 10 min at room temperature for 5 min. This procedure was repeated three times. Finally, *Streptococcus mutans*, *Actinomyces viscosus*, *Porphyromonas gingivalis*, and *Enterococcus faecalis* were prepared to a bacteria solution at 0.5% McFarland concentration and bacterial concentration of 1.5 × 10^8^ CFU/mL [34] through a McFarland turbidimeter (Densicheck, France BioMérieux), respectively. The solutions were used in the next stage.

### 2.4. Quality Control and Prevention of Bias

The methods of quality control and prevention of bias were as follows: (1) A unified solidification standard and an equipment inspection method were used to inspect the material model disks clinically. (2) The preparation methods and solidification time of each material model block were strictly standardized. (3) All the sealing material disks were prepared by the same person to reduce system error.

#### 2.4.1. The Adhesion Ability of Different Bacteria to Four Dental Filling Materials and Five Root Canal Sealers

Three disinfected dental restorative materials and five root canal filling material model disks were placed in 48-well plates. The three dental restorative materials were divided into two groups: *Streptococcus mutans* and *Actinomyces viscosus*; the five root canal sealers were divided into two groups: *Porphyromonas gingivalis* and *Enterococcus faecalis*. In the *Streptococcus mutans* group, 2 mL of BHI liquid medium and 100 *μ*L of *Streptococcus mutans* suspension (1.5 × 10^8^ CFU/mL) were added. In the *Actinomyces viscosus* group, 2 mL of BHI liquid medium and 100 *μ*L of *Actinomyces viscosus* suspension (1.5 × 10^8^ CFU/mL) were added to the culture under anaerobic conditions equilibrated in an atmosphere consisting of 10% CO_2_, 10% H_2_, and 80% N_2_ at 37°C for 2, 4, 6, 8, 12, 16, 20, and 24 h, respectively [35]. Each dental material model block received 2 mL of BHI liquid medium and 100 µL PBS as the control group. After incubation for the specified periods, each well was washed twice with PBS, and then, glutaraldehyde (diluted 10 times) was added for fixing for 30 min, followed by washing. In the *Porphyromonas gingivalis* group, 2 mL of BHI liquid medium and 100 *μ*L of *Porphyromonas gingivalis* suspension (1.5 × 10^8^ CFU/mL) were added. In the *Enterococcus faecalis* group, 2 mL of BHI liquid medium and 100 µL of *Enterococcus suspension* (1.5 × 10^8^ CFU/mL) were added to the culture at 37°C for 2, 4, 6, 8, 12, 16, 20, 24 h under anaerobic conditions [35]. After incubation for the specified periods, each well was washed twice with PBS, and then, glutaraldehyde (diluted 10 times) was added for fixing for 30 min, followed by washing.  Table 2 presents the specific grouping, the processing methods, and time intervals in each group. The dental material model block assay was repeated three times for each bacterial species. Concentrations of bacterial cells on each material model block samples were measured by colony counting.

#### 2.4.2. Secondary Electron Mode Observation through Field Emission Scanning Electron Microscopy (FESEM)

The material model block samples of the above groups were sequentially dehydrated with 37.5%, 50%, 70%, 90%, and 100% ethanol solution gradients (each gradient was used to dehydrate for 10 minutes, and 100% ethanol solution was used twice). Finally, the samples were immersed in hexamethyldisilazide (HMDS) three times (15 minutes each time) and dried at room temperature. The bacteria on the sample surfaces were observed and photographed through FESEM (SU-70, Hitachi, Japan) under secondary electron mode. Before FESEM observations, the samples were sprayed with gold for 60 seconds (E-1020). Image-Pro Plus 16.0 (IPP) image processing and analysis software (USA) was employed for image analysis to calculate the number of different bacteria adhering to various material model blocks and compare the bacterial counts adhering to various materials at different time intervals [35].

### 2.5. Statistical Analysis

SPSS 16.0 was adopted for statistical analyses. The data were expressed as the mean ± standard deviation (*x* ± *s*), and comparisons between the multiple groups of independent, normal, and equal variance measurement data were carried out using multi-factor analysis of variance. One-way ANOVA was used to compare differences between the groups. Dunnett's two-sided *t*-test was employed to analyze the differences between the experimental and control groups. *P* < 0.05 was considered a significant difference.

## 3. Results

The experimental results were expressed as mean ± standard deviation (unit: ×10^6^ CFU/ml; number/×5000 visual field).

### 3.1. *Streptococcus mutans* Group

**Figure 1:**
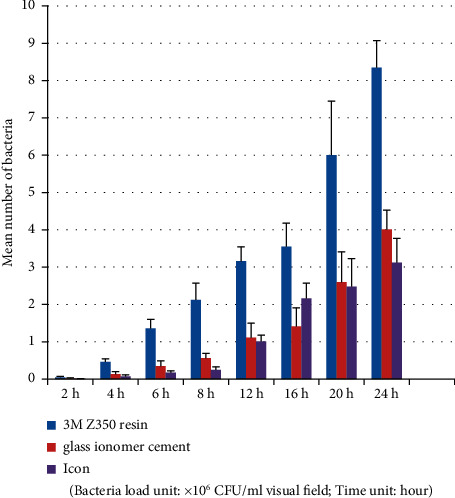
*Streptococcus mutans* group (plate colony counting).

**Figure 2:**
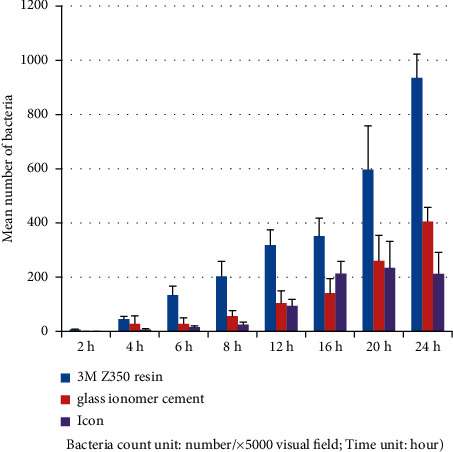
*Streptococcus mutans* group (electron microscopy counting).

**Figure 3:**
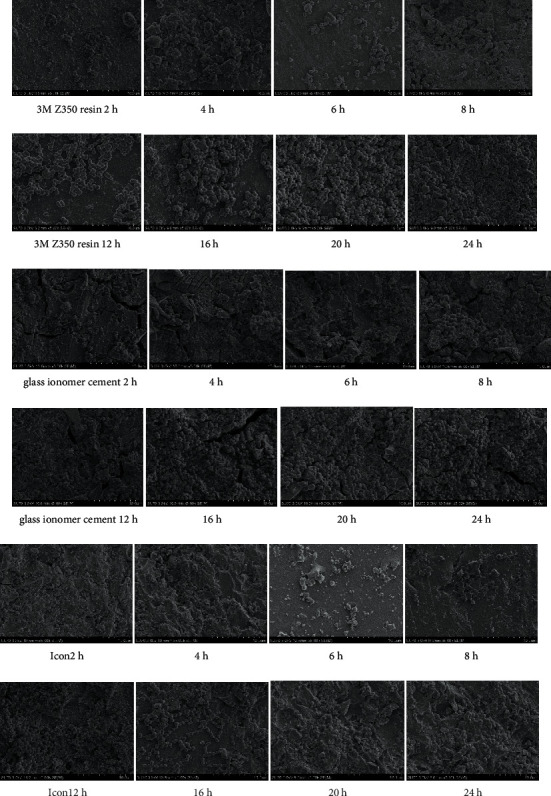
Electron microscopy pictures of the *Streptococcus mutans* group.

Tables 3 and 4 and Figures 1–3 show that in the *Streptococcus mutans* experimental group, the number of bacteria on the resin blocks was significantly higher than on the glass ionomer and icon blocks (*P* < 0.05). However, there was no significant difference in the number of bacteria adhering to the glass ionomer and icon blocks (*P* > 0.05). The results of bacterial plate counts were consistent with those of electron microscopy. Therefore, the adhesion ability of *Streptococcus mutans* to the three material blocks after 24 h was ranked as follows: resin > glass ionomer > icon. A comparison of time intervals showed that at the 12-hour interval, the number of bacteria adhering to each material block increased significantly (*P* < 0.05).

### 3.2. *Actinomyces viscosus* Group

**Figure 4:**
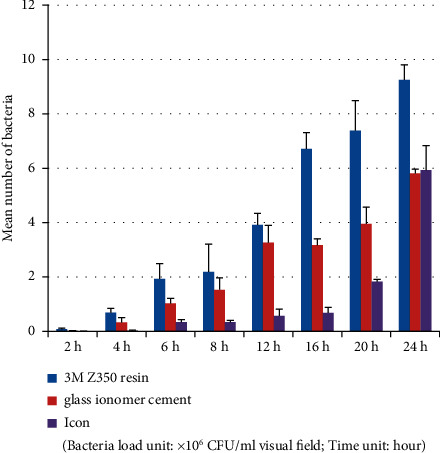
*Actinomyces viscosus* group (plate colony counting).

**Figure 5:**
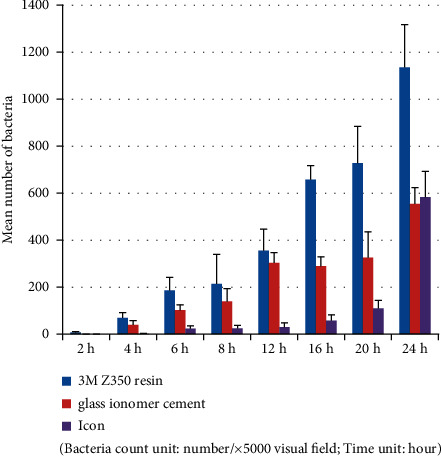
*Actinomyces viscosus* group(electron microscopy counting).

**Figure 6:**
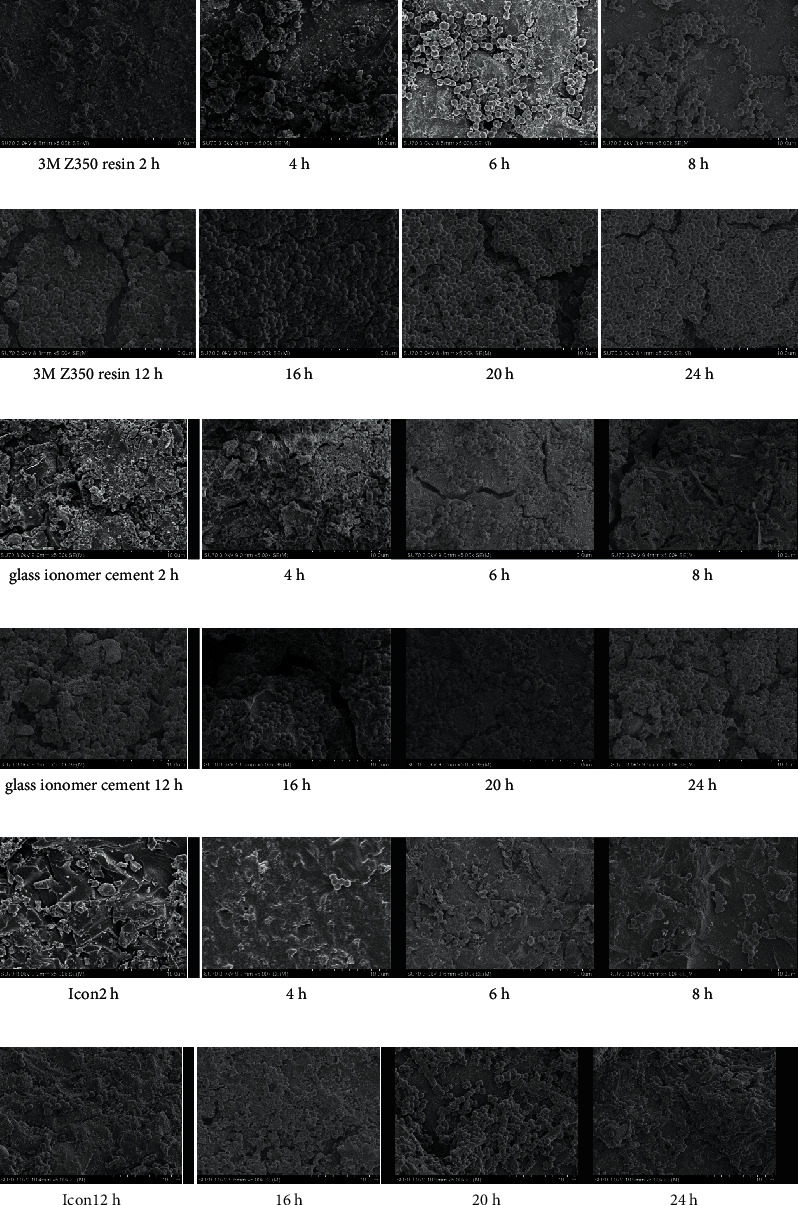
Electron microscopy pictures of the *Actinomyces viscosus* group.

Tables 5 and 6 and Figures 4–6 show that in the *Actinomyces viscosus* experimental group, the bacteria exhibited maximum adhesion to the resin blocks (*P* < 0.05), similar to the *Streptococcus mutans* group. Unlike the *Streptococcus mutans* group, the number of bacteria on the icon blocks was significantly lower than on the glass ionomer blocks during the first 24 hours (*P*<0.05). However, after 24 hours, the number of bacteria on icon blocks slightly outnumbered that of glass ionomer blocks. Therefore, the adhesion ability of *Actinomyces viscosus* to the four material blocks after 24 hours was ranked as follows: resin > icon > glass ionomer. The bacterial plate counts were the same as those of electron microscopy. A comparison of time intervals showed that at the 8-hour interval, there were significant increases in the number of bacteria adhering to the material blocks (*P* < 0.05).

### 3.3. *Porphyromonas gingivalis* Group

**Figure 7:**
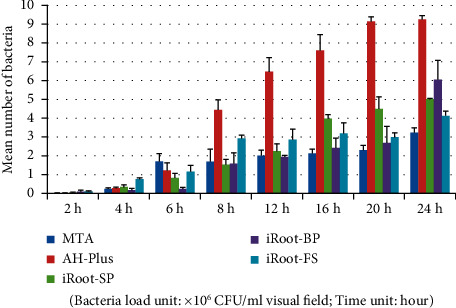
*Porphyromonas gingivalis* group (plate colony counting).

**Figure 8:**
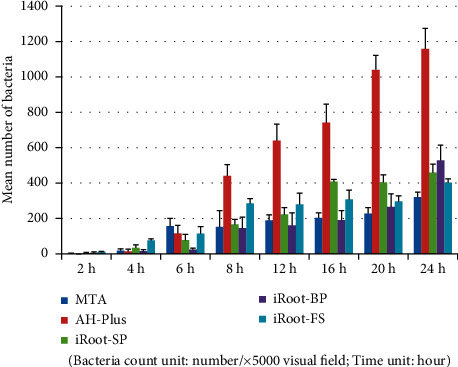
*Porphyromonas gingivalis* group (electron microscopy counting).

**Figure 9:**
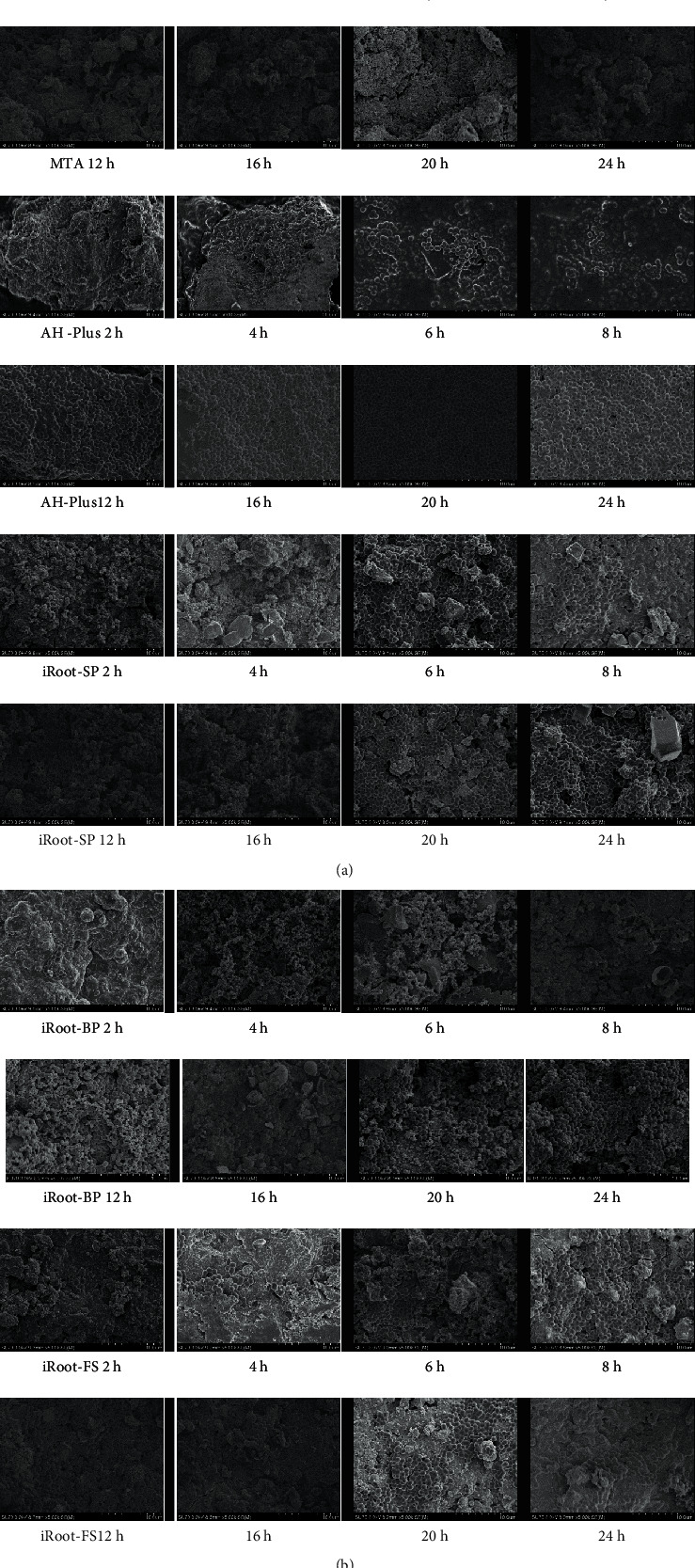
Electron microscopy pictures of the *Porphyromonas gingivalis* group.

Tables 7 and 8 and Figures 7–9 show that in the *Porphyromonas gingivalis* group, the bacterial load on the AH-Plus blocks was significantly higher than the other four root canal material blocks (*P* < 0.05). A comparison of adhering bacterial counts between the MTA, iRoot-SP, iRoot-BP, and iRoot-FS blocks revealed no significant differences (*P* > 0.05). Therefore, the adhesion ability of *Porphyromonas gingivalis* to five material blocks after 24 hours was ranked as follows: AH-Plus > iRoot-BP > iRoot-SP > iRoot-FS > MTA. The electron microscope observations also revealed similar results. A comparison of time intervals showed a significant increase in the number of bacteria adhering to each material block at the 8-hour interval (*P* < 0.01).

### 3.4. *Enterococcus faecalis* Group

**Figure 10:**
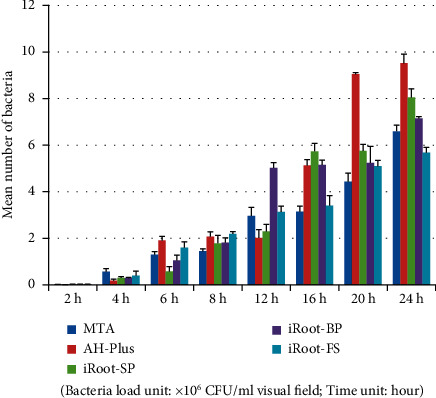
*Enterococcus faecalis* group (plate colony counting).

**Figure 11:**
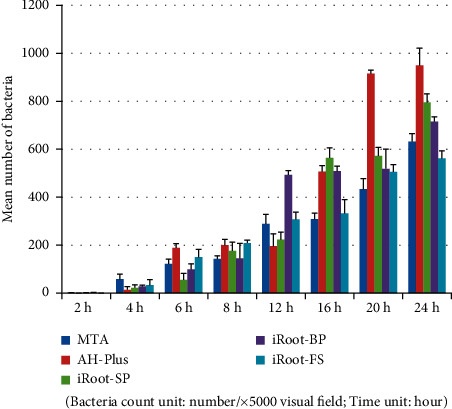
*Enterococcus faecalis* group (electron microscopy counting).

**Figure 12:**
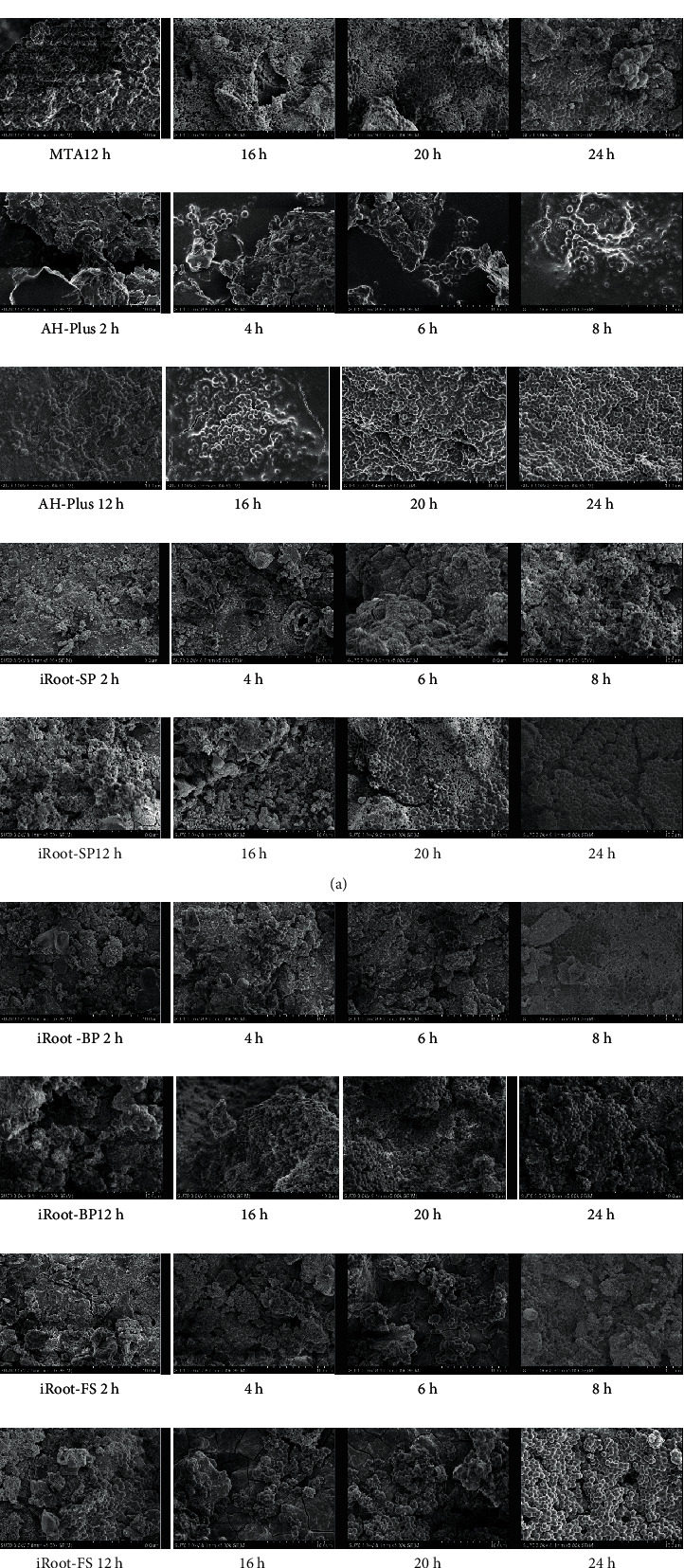
Electron microscopy pictures of the *Enterococcus faecalis* group.

Tables 9 and 10 and Figures 10–12 show that in the *Enterococcus faecalis* experimental group, there was no significant difference in bacterial adhesion between the MTA and iRoot-FS blocks (*P* > 0.05). A comparison of bacterial adhesion between the MTA, AH-Plus, iRoot-SP, and the iRoot-BP blocks showed significantly lower MTA load (*P* < 0.05). A comparison of bacterial adhesion between the AH-Plus, iRoot-SP, and iRoot-BP blocks revealed no significant differences (*P* > 0.05), consistent with the electron microscopy results. Therefore, the adhesion ability of *Enterococcus faecalis* to five material blocks after 24 hours was ranked as follows: AH-Plus > iRoot-SP > iRoot-BP > MTA > iRoot-FS. A comparison of time intervals showed a significant increase in the number of bacteria adhering to each material block at the 6-hour interval (*P* < 0.05).

## 4. Discussion

This study investigated the differences in the adhesion properties of supragingival and subgingival plaque to different dental restorative materials and root canal sealers *in vitro* and verified the hypothesis we mentioned before. In addition, the differences in the adhesion properties of different bacterial species to different materials and the biofilm formation were evaluated at different time intervals to provide a basis to select dental materials and help researchers develop novel dental materials. Many studies have evaluated the adhesion of different bacterial species to dental materials; however, relatively few studies have included different time intervals in the study design, which is an advantage of the present study. A comparison of time intervals showed that at different time intervals, the number of bacteria adhering to each material block increased significantly or not and provided a theoretical basis for the selection of antibacterial properties and antibacterial time of new materials in the future development. Also, provided the guidance on how to care for filling materials in the mouth.

According to the results, *Streptococcus mutans* and *Actinomyces viscosus* exhibited the strongest adhesion ability to the resin material blocks. In contrast, the adhesion ability of *Streptococcus mutans* and *Actinomyces viscosus* to the glass ionomer and the icon material blocks was lower than to the resin material blocks. There was no significant difference in the number of bacteria adhering to the glass ionomer and icon blocks.

Composite resins have good aesthetics, good mechanical properties, and high biosafety. They have gone through the process of changing from the chemical curing type to light curing type, from a large particle filler to nano-mixed filler, and their performance has been continuously improved. However, they also have certain defects in clinical application, and secondary caries are one of the main reasons for the failure of restoration. The main reason for secondary caries is the inevitable volume shrinkage of resin monomers during the polymerization process, which leads to the formation of microleakage between the resin and the dental tissue, and the penetration of various microorganisms into the cracks. A large number of experiments have confirmed that BIS-GMA, triethylene glycol dimethacrylate (TEGDMA), urethane dimethacrylate, and silica inorganic filler in the resin system have no inhibitory effect on cariogenic bacteria.

Glass ionomer cement contains lanthanum calcium fluoroaluminate glass and can release fluoride ions. Fluoride release can be achieved by infiltration of water-soluble salts, fluoride-releasing filler systems, or matrix-bound fluoride forms [21]; fluoride release is stimulated by generating 2-hydroxyethyl methacrylate to increase the hydrophilicity of the matrix [36]. The release of fluoride from composite resins is less than that of glass ionomers and gradually decreases over time [37]. However, there might be discrepancies in the data reported by clinical studies on the fluoride-releasing materials that can significantly prevent secondary caries and affect the growth of caries-related bacteria [21]. Similar to studies on antibiotic-releasing systems, these contradictory results might be attributed to different ratios of the released material to the fluid volume, leading to the washing away of released antibiotics. Another effective way to increase the antimicrobial properties of materials is to fix antimicrobial components on the surface of biological materials to maintain their antimicrobial efficacy. Therefore, it is possible to incorporate antibacterial ingredients into composite resins to modify them so that the bacteria will be inactivated when they contact the modified resin, inhibiting biofilm formation [38–40]. However, further observations are necessary to determine whether these modifications can significantly affect dynamic clinical conditions [41].

The surface properties of materials, such as surface roughness and surface free energy, may have a decisive influence on bacterial adhesion. Materials with high surface roughness and low hydrophobicity are more likely to accumulate plaque than materials with low surface roughness and high hydrophobicity [42]. Traditional composite resins, such as 3M Z350, are mainly composed of a methacrylate-based resin matrix mixed with fillers of different particle sizes. The infiltrating resin icon is mainly composed of a methyl methacrylate resin matrix without filler particles and has high fluidity. The polished icon can form a very smooth and flat surface, while the surface of other materials is rougher, with more scattered small pores, with particles detached from the surface. Differences in the properties and surface roughness of materials may be the main factors affecting the differences in bacterial adhesion. Therefore, improving the smoothness of the material surface without affecting its mechanical properties has also become a research focus to reduce the bacterial adhesion rate on the dental material surface.

According to the results*, Porphyromonas gingivalis* and *Enterococcus faecalis* exhibited the highest adhesion ability to the AH-Plus sealer block, while the adhesion ability of *Porphyromonas gingivalis* to MTA was the lowest. *Enterococcus faecalis* exhibited the lowest adhesion ability to iRoot-FS.

Concerning root canal sealing materials, recently, popular bioceramic materials, such as the iRoot series and MTA, have attracted attention due to their excellent biocompatibility and antibacterial properties. This study showed that AH-Plus, the classic root canal sealer material, had poor antibacterial performance, which might explain the failure of some root canal treatments. AH-Plus is an epoxy resin root canal sealer, the antibacterial effect of this type of sealant is mainly derived from the bisphenol A-diglycidyl ether and formaldehyde components released during the curing process. However, as the curing of root canal sealers, less and less antibacterial components are released, so their antibacterial effect will gradually diminish or even be completely lost within hours to days. iRoot series materials belong to calcium silicate root canal sealers; they mainly have a bactericidal effect on bacteria through the destruction of cell membrane or DNA and protein denaturation by Ca(OH)_2_. Its antibacterial effect is stronger than AH-Plus. In particular, the iRoot series materials have good biocompatibility and easy handling. This provides us with a theoretical basis for selecting materials according to the characteristics of the case in endodontic treatment.

This study showed that resin and AH-Plus sealer, currently the most commonly used dental materials in China, exhibited the highest bacterial adhesion and were the easiest to form biofilms. It was found that although there were many kinds of dental materials and had different degrees of the antibacterial effect, their antibacterial effect was weak and the duration was short. Up to now, it is still a daunting challenge to synthesize oral restorative materials and root canal sealers with long-term, stable, and effective bactericidal effects and good biocompatibility, and it is also the future direction of research on endodontic treatment. The aim of this study is to reduce the occurrence of secondary caries, improve the success rate of root canal therapy, maintain the long-term efficacy of root canal therapy and prevent root canal therapy failure to provide new ideas and theoretical support. It has recently become a research hotspot to determine how to change the surface properties of materials and reduce the adhesion of bacteria and biofilm formation. Icon and glass ionomer have a certain antibacterial effect because of their chemical properties, smooth surface, and fluoride release, but their low strength decreases clinical applications. It has become an urgent research goal to determine how to combine the respective advantages of these materials to develop antibacterial properties without affecting the surface roughness and strength of the material to reduce secondary caries and the incidence of filler shedding.

Due to the limited objective conditions and practical research level, there was a limitation in this research that we have not designed the animal experiments yet. We would keep it up to do further animal work to verify these theories in the next stage.

## Figures and Tables

**Table 1:** The composition of the dental materials.

Dental material	Brand	Origin	Composition
3M Z350 resin	3M ESPE	USA	Silanized ceramic, silanized zirconia silica, dimethacrylate, bisphenol A-diglycidyl ether dimethacrylate, and ethoxylated bisphenol A dimethacrylate polydiester

Glass ionomer cement	3M ESPE KetacTM Molar Easymix	USA	Lanthanum calcium fluoroaluminate glass, acrylic acid, and maleic acid

Icon penetration resin	DMG	Germany	Hydrochloric acid, pyrosilicic acid, ethanol, methyl methacrylate resin matrix, and surface active substances

MTA	DENTSPLY international. Inc	USA	Trioxy mineral polymer, calcium silicate, calcium phosphate, and calcium oxide

iRoot-SP	Innovative BioCeramix inc	Canada	Zirconia, calcium silicate, calcium hydroxide, and calcium dihydrogen phosphate

iRoot-BP	Innovative BioCeramix inc	Canada	Zirconia, calcium silicate, tantalum oxide, calcium dihydrogen phosphate, solidifying agent, and filler

iRoot-FS	Brasseler	USA	Zirconia, calcium silicate, tantalum oxide, calcium dihydrogen phosphate, solidifying agent, and filler

AH-plus	DENTSPLY detrey GmbH	German	Bisphenol A epoxy resin, bisphenol F epoxy resin, calcium tungstate, zirconium oxide, silicon, iron oxide, diphenyldiamine, aminoadamantane, and tricyclodecane diamine

**Table 2:** The group of dental material model blocks and the processing time.

Group	Dental material model blocks
*Streptococcus mutans* group	1. 3M Z350 resin 2 h, 4 h, 6 h, 8 h, 12 h, 16 h, 20 h, 24 h
	2. Glass ionomer cement 2 h, 4 h, 6 h, 8 h, 12 h, 16 h, 20 h, 24 h
	3. Icon 2 h, 4 h, 6 h, 8 h, 12 h, 16 h, 20 h, 24 h

*Actinomyces viscosus* group	1. 3M Z350 resin 2 h, 4 h, 6 h, 8 h, 12 h, 16 h, 20 h, 24 h
	2. Glass ionomer cement 2 h, 4 h, 6 h, 8 h, 12 h, 16 h, 20 h, 24 h
	3. Icon 2 h, 4 h, 6 h, 8 h, 12 h, 16 h, 20 h, 24 h

*Porphyromonas gingivalis* group	1. MTA 2 h, 4 h, 6 h, 8 h, 12 h, 16 h, 20 h, 24 h
	2. iRoot-SP 2 h, 4 h, 6 h, 8 h, 12 h, 16 h, 20 h, 24 h
	3. iRoot-BP 2 h, 4 h, 6 h, 8 h, 12 h, 16 h, 20 h, 24 h
	4. iRoot-FS 2 h, 4 h, 6 h, 8 h, 12 h, 16 h, 20 h, 24 h
	5. AH-plus 2 h, 4 h, 6 h, 8 h, 12 h, 16 h, 20 h, 24 h

*Enterococcus faecalis* group	1. MTA 2 h, 4 h, 6 h, 8 h, 12 h, 16 h, 20 h, 24 h
	2. iRoot-SP 2 h, 4 h, 6 h, 8 h, 12 h, 16 h, 20 h, 24 h
	3. iRoot-BP 2 h, 4 h, 6 h, 8 h, 12 h, 16 h, 20 h, 24 h
	4. iRoot-FS 2 h, 4 h, 6 h, 8 h, 12 h, 16 h, 20 h, 24 h
	5. AH-plus 2 h, 4 h, 6 h, 8 h, 12 h, 16 h, 20 h, 24 h

**Table 3:** *Streptococcus mutans* group (plate colony counting).

	2 h	4 h	6 h	8 h	12 h	16 h	20 h	24 h
3M Z350 resin	**0.05** **±** **0.02**	**0.46** **±** **0.08**	**1.36** **±** **0.24**	**2.12** **±** **0.45**	**3.16** **±** **0.38**	**3.55** **±** **0.63**	**6.00** **±** **1.45**	**8.35** **±** **0.72**
Glass ionomer cement	**0.02** **±** **0.01**	**0.13** **±** **0.07**	**0.35** **±** **0.14**	**0.56** **±** **0.13**	**1.11** **±** **0.39**	**1.41** **±** **0.50**	**2.60** **±** **0.81**	**4.01** **±** **0.52**
Icon	**0.01** **±** **0.00**	**0.07** **±** **0.04**	**0.17** **±** **0.05**	**0.25** **±** **0.08**	**1.01** **±** **0.17**	**2.16** **±** **0.41**	**2.48** **±** **0.75**	**3.12** **±** **0.65**

The experimental results were expressed as mean ± standard deviation (unit: ×10^6^ CFU/ml).

**Table 4:** *Streptococcus mutans* group (electron microscopy counting).

	2 h	4 h	6 h	8 h	12 h	16 h	20 h	24 h
3M Z350 resin	**6.00** **±** **2.00**	**45.33** **±** **9.71**	**133.67** **±** **33.53**	**202.67** **±** **55.63**	**318.00** **±** **56.24**	**351.67** **±** **65.96**	**597.00** **±** **161.61**	**935.33** **±** **87.89**

Glass ionomer cement	**0** **±** **0**	**28.00** **±** **29.51**	**27.67** **±** **22.14**	**56.33** **±** **19.86**	**104.33** **±** **44.97**	**140.67** **±** **53.72**	**259.67** **±** **94.56**	**404.67** **±** **53.26**

Icon	**0** **±** **0**	**6.33** **±** **3.51**	**15.67** **±** **5.03**	**24.67** **±** **9.71**	**93.67** **±** **24.79**	**212.67** **±** **45.36**	**234.67** **±** **97.60**	**212.00** **±** **79.61**

The experimental results were expressed as mean ± standard deviation (unit: number/×5000 visual field).

**Table 5:** *Actinomyces viscosus* group (plate colony counting).

	2 h	4 h	6 h	8 h	12 h	16 h	20 h	24 h
3M Z350 resin	**0.08 ± 0.04**	**0.69 ± 0.15**	**1.93 ± 0.56**	**2.19 ± 1.02**	**3.92 ± 0.42**	**6.71 ± 0.60**	**7.38 ± 1.11**	**9.25 ± 0.55**
Glass ionomer cement	**0.02 ± 0.01**	**0.33 ± 0.17**	**1.03 ± 0.18**	**1.53 ± 0.44**	**3.26 ± 0.64**	**3.17 ± 0.23**	**3.95 ± 0.62**	**5.81 ± 0.16**
Icon	**0.01 ± 0.00**	**0.03 ± 0.01**	**0.34 ± 0.09**	**0.34 ± 0.06**	**0.57 ± 0.25**	**0.68 ± 0.20**	**1.83 ± 0.08**	**5.93 ± 0.90**

The experimental results were expressed as mean ± standard deviation (unit: ×10^6^ CFU/ml).

**Table 6:** *Actinomyces viscosus* group (electron microscopy counting).

	2 h	4 h	6 h	8 h	12 h	16 h	20 h	24 h
3M Z350 resin	**7.33 ± 4.16**	**69.00 ± 22.27**	**186.33 ± 55.90**	**214.67 ± 124.98**	**356.00 ± 91.028**	**657.67 ± 60.14**	**728.33 ± 155.52**	**1135.00 ± 182.59**
Glass ionomer cement	**0 ± 0**	**39.67 ± 17.62**	**102.00 ± 22.61**	**140.00 ± 54.25**	**303.00 ± 44.58**	**290.00 ± 39.23**	**325.33 ± 109.95**	**554.33 ± 68.92**
Icon	**0 ± 0**	**1.33 ± 2.31**	**24.00 ± 11.14**	**25.00 ± 12.77**	**29.67 ± 18.82**	**57.33 ± 24.50**	**110.00 ± 34.04**	**583.00 ± 109.67**

The experimental results were expressed as mean ± standard deviation (unit: number/×5000 visual field).

**Table 7:** *Porphyromonas gingivalis* group (plate colony counting).

	2 h	4 h	6 h	8 h	12 h	16 h	20 h	24 h
MTA	**0.02** **±** **0.01**	**0.24** **±** **0.06**	**1.70** **±** **0.41**	**1.69** **±** **0.66**	**2.01** **±** **0.29**	**2.13** **±** **0.23**	**2.30 ± 0.25**	**3.23** **±** **0.25**
AH-plus	**0.02** **±** **0.01**	**0.28** **±** **0.05**	**1.22** **±** **0.40**	**4.44** **±** **0.53**	**6.47** **±** **0.75**	**7.61** **±** **0.84**	**9.15** **±** **0.24**	**9.26 ± 0.20**
iRoot-SP	**0.04** **±** **0.03**	**0.34** **±** **0.11**	**0.83** **±** **0.23**	**1.53** **±** **0.28**	**2.25** **±** **0.38**	**3.98** **±** **0.21**	**4.50** **±** **0.64**	**5.02 ± 0.04**
iRoot –BP	**0.09** **±** **0.09**	**0.18** **±** **0.09**	**0.24** **±** **0.08**	**1.58** **±** **0.58**	**1.94** **±** **0.08**	**2.42** **±** **0.52**	**2.69** **±** **0.88**	**6.05 ± 1.02**
iRoot –FS	**0.11** **±** **0.03**	**0.76** **±** **0.06**	**1.16** **±** **0.33**	**2.92** **±** **0.18**	**2.86** **±** **0.56**	**3.19** **±** **0.56**	**2.99** **±** **0.23**	**4.13 ± 0.25**

The experimental results were expressed as mean ± standard deviation (unit: ×10^6^ CFU/ml).

**Table 8:** *Porphyromonas gingivalis* group (electron microscopy counting).

	2 h	4 h	6 h	8 h	12 h	16 h	20 h	24 h
MTA	**1.33** **±** **2.31**	**17.33** **±** **10.60**	**156.33** **±** **44.11**	**152.67** **±** **91.96**	**189.33** **±** **30.01**	**203.00** **±** **27.62**	**226.67 ± 34.53**	**320.00** **±** **28.48**
AH-plus	**0** **±** **0**	**14.00** **±** **12.77**	**115.00** **±** **44.93**	**441.00** **±** **62.98**	**640.33** **±** **93.38**	**741.33** **±** **104.53**	**1040.70** **±** **81.13**	**1159.00 ± 114.97**
iRoot-SP	**4.00** **±** **4.00**	**33.67** **±** **17.24**	**77.00** **±** **32.51**	**166.00** **±** **28.58**	**222.00** **±** **39.13**	**407.67** **±** **13.43**	**403.67** **±** **42.59**	**458.33 ± 48.58**
iRoot –BP	**5.33** **±** **5.51**	**14.67** **±** **8.74**	**23.67** **±** **8.02**	**145.00** **±** **62.23**	**161.00** **±** **70.70**	**190.00** **±** **54.44**	**265.67** **±** **73.28**	**528.33 ± 85.54**
iRoot –FS	**10.00** **±** **4.00**	**76.33** **±** **8.33**	**114.00** **±** **39.04**	**285.33** **±** **26.10**	**279.00** **±** **64.55**	**307.00** **±** **53.25**	**296.67** **±** **31.02**	**402.00 ± 21.93**

The experimental results were expressed as mean ± standard deviation (unit: number/×5000 visual field).

**Table 9:** *Enterococcus faecalis* group (plate colony counting).

	2 h	4 h	6 h	8 h	12 h	16 h	20 h	24 h
MTA	**0.02** **±** **0.00**	**0.57** **±** **0.13**	**1.30** **±** **0.13**	**1.45** **±** **0.10**	**2.96** **±** **0.37**	**3.15** **±** **0.23**	**4.44** **±** **0.36**	**6.60** **±** **0.26**
AH-plus	**0.01** **±** **0.00**	**0.18** **±** **0.07**	**1.91** **±** **0.17**	**2.07** **±** **0.21**	**2.02** **±** **0.36**	**5.13** **±** **0.25**	**9.05** **±** **0.06**	**9.52** **±** **0.39**
iRoot –SP	**0.02** **±** **0.01**	**0.30** **±** **0.06**	**0.58** **±** **0.21**	**1.79** **±** **0.34**	**2.30** **±** **0.30**	**5.74** **±** **0.34**	**5.76** **±** **0.27**	**8.05** **±** **0.37**
iRoot –BP	**0.02** **±** **0.01**	**0.29** **±** **0.03**	**1.05** **±** **0.23**	**1.82** **±** **0.20**	**5.03** **±** **0.22**	**5.15** **±** **0.21**	**5.24** **±** **0.71**	**7.15** **±** **0.08**
iRoot –FS	**0.03** **±** **0.01**	**0.40** **±** **0.19**	**1.60** **±** **0.25**	**2.19** **±** **0.10**	**3.14** **±** **0.24**	**3.40** **±** **0.44**	**5.10** **±** **0.25**	**5.68** **±** **0.23**

The experimental results were expressed as mean ± standard deviation (unit: ×10^6^ CFU/ml).

**Table 10:** *Enterococcus faecalis* group (electron microscopy counting).

	2 h	4 h	6 h	8 h	12 h	16 h	20 h	24 h
MTA	**0.67** **±** **1.15**	**58.67** **±** **20.84**	**121.67** **±** **20.31**	**142.67** **±** **12.90**	**289.00** **±** **39.61**	**308.00** **±** **25.24**	**433.33** **±** **44.50**	**631.33** **±** **33.25**
AH-plus	**0** **±** **0**	**14.00** **±** **12.77**	**189.00** **±** **17.78**	**199.67** **±** **24.17**	**196.00** **±** **50.51**	**506.67** **±** **24.78**	**915.00** **±** **14.45**	**949.33** **±** **72.39**
iRoot –SP	**0.67** **±** **1.15**	**22.33** **±** **11.68**	**55.33** **±** **27.47**	**175.67** **±** **37.31**	**222.67** **±** **31.56**	**564.33** **±** **41.68**	**572.67** **±** **35.44**	**795.00** **±** **35.68**
iRoot –BP	**1.00** **±** **1.73**	**28.33** **±** **5.03**	**98.67** **±** **22.90**	**145.00** **±** **62.55**	**492.67** **±** **17.62**	**508.33** **±** **21.20**	**518.67** **±** **81.28**	**715.33** **±** **19.22**
iRoot –FS	**0** **±** **0**	**33.33** **±** **22.59**	**149.67** **±** **32.96**	**209.00** **±** **12.12**	**307.67** **±** **29.74**	**333.00** **±** **57.09**	**506.00** **±** **30.05**	**561.67** **±** **30.99**

The experimental results were expressed as mean ± standard deviation (unit: number/×5000 visual field).

## Data Availability

The datasets generated during and/or analysed during the current study are available from the corresponding author on reasonable request.

## Abbreviations

*Sm*: *Streptococcus mutans*
*Pg*: *Porphyromonas gingivalis*
*Av*: *Actinomyces viscosus*
*Ef*: *Enterococcus faecalis*
GIC: Glass ionomer cement
MTA: Mineral trioxide aggregate
iRoot-SP: Injectable root canal sealer-sealing paste
iRoot-BP: iRoot -bioaggregate putty
iRoot-FS: iRoot-fast set putty.
